# Structure, function, and immunomodulation of the CD8 co-receptor

**DOI:** 10.3389/fimmu.2024.1412513

**Published:** 2024-08-26

**Authors:** Shreyaa Srinivasan, Cheng Zhu, Andrew C. McShan

**Affiliations:** ^1^ School of Chemistry and Biochemistry, Georgia Institute of Technology, Atlanta, GA, United States; ^2^ Wallace H. Coulter Department of Biomedical Engineering, Georgia Institute of Technology and Emory University, Atlanta, GA, United States; ^3^ George W. Woodruff School of Mechanical Engineering, Georgia Institute of Technology, Atlanta, GA, United States

**Keywords:** CD8 co-receptor, immunomodulation, T cell signaling, T cell receptor, major histocompatibility complex, monoclonal antibodies, chimeric antigen receptor

## Abstract

Expressed on the surface of CD8^+^ T cells, the CD8 co-receptor is a key component of the T cells that contributes to antigen recognition, immune cell maturation, and immune cell signaling. While CD8 is widely recognized as a co-stimulatory molecule for conventional CD8^+^ αβ T cells, recent reports highlight its multifaceted role in both adaptive and innate immune responses. In this review, we discuss the utility of CD8 in relation to its immunomodulatory properties. We outline the unique structure and function of different CD8 domains (ectodomain, hinge, transmembrane, cytoplasmic tail) in the context of the distinct properties of CD8αα homodimers and CD8αβ heterodimers. We discuss CD8 features commonly used to construct chimeric antigen receptors for immunotherapy. We describe the molecular interactions of CD8 with classical MHC-I, non-classical MHCs, and Lck partners involved in T cell signaling. Engineered and naturally occurring CD8 mutations that alter immune responses are discussed. The applications of anti-CD8 monoclonal antibodies (mABs) that target CD8 are summarized. Finally, we examine the unique structure and function of several CD8/mAB complexes. Collectively, these findings reveal the promising immunomodulatory properties of CD8 and CD8 binding partners, not only to uncover basic immune system function, but to advance efforts towards translational research for targeted immunotherapy.

## 1 Introduction

The immune system is a complex network of molecular interactions and cellular responses many of which involve and/or depend on the function of cells expressing either CD4 or CD8 co-receptors on their surfaces (1, 2). CD4 expresses primarily on the surface of helper T cells whereas CD8 expresses on the surface of cytotoxic and suppressor T cells (3). CD8^+^ T cells detect pathogens, cancer, and autoimmunity towards eliminating diseased cells through T cell antigen receptor (TCR) recognition of antigens presented by classical and non-classical major histocompatibility complex class I (MHC-I) molecules on the surface of all nucleated cells (4). CD4 and CD8 are termed co-receptors because they bind the same MHC ligand as the receptor TCR. However, CD4 and CD8 associate with the membrane-proximal domains of the MHC-II and I molecules, respectively, as opposed to the TCR, which binds the membrane-distal domains of the MHC-II and I molecules, respectively (5, 6). CD8 is a dimeric receptor that contains several domains: ectodomain, hinge, transmembrane, and cytoplasmic tail (7). CD8 primarily acts as a co-stimulator, and occasionally as a co-repressor, of immune responses (7, 8). Its mechanism of action is thought to occur through several cooperative events, each differing based on the cell type and the immunoreceptors involved. Firstly, the association of CD8 with MHC-I stabilizes TCR/antigen/MHC-I complexes (9–13). High affinity TCR/antigen/MHC-I interactions (K_D_ < 10 μM) can result in CD8^+^ T cell activation without the need for CD8 (7, 14, 15). However, in cases of low to medium affinity TCR/antigen/MHC-I interactions (K_D_ > 30 μM), CD8 stabilizes the TCR/antigen/MHC-I complex to enhance recognition stability, sensitivity, and specificity (7, 14, 15). CD8 also participates in immune cell mechanotransduction by promoting dynamic catch bonds that result from the cooperativity between TCR/CD8/antigen/MHC-I interactions (16). It should be noted that several groups have identified non-canonical TCR/MHC docking modes for which some conformations could still accommodate CD8 binding to the MHC (17–20). However, several pieces of evidence suggest some of these non-canonical binding modes (i.e., reverse polarity TCR binding) would not allow for robust CD8 interaction with the MHC (21–23). Secondly, the cytoplasmic tail of CD8 binds and recruits the lymphocyte-specific protein tyrosine kinase, p56^Lck^ (Lck), to the TCR/CD3 complex. Here, Lck helps in initiating TCR signaling by phosphorylating immunoreceptor tyrosine-based activation motifs (ITAMs) located within the cytoplasmic tails of CD3γ, CD3δ, CD3ϵ, and CD3ζ subunits associated with the TCR (24–26). The phosphorylated ITAMs serve as docking sites for another kinase, ZAP-70, which phosphorylates downstream signaling proteins, Linker for activation of T cells (LAT) and SLP-76, ultimately resulting in the release of cytokines, granzymes, and perforin towards the target cell (27, 28). Beyond its classical role in CD8^+^ cytotoxic T cell signaling, CD8 has also demonstrated contributions to T cell development/maturation, T cell differentiation, immune responses in a wide range of unconventional immune cell subsets, and cross-talk with B cell mediated responses (several of these are discussed in section 2.1).

**Figure 1:**
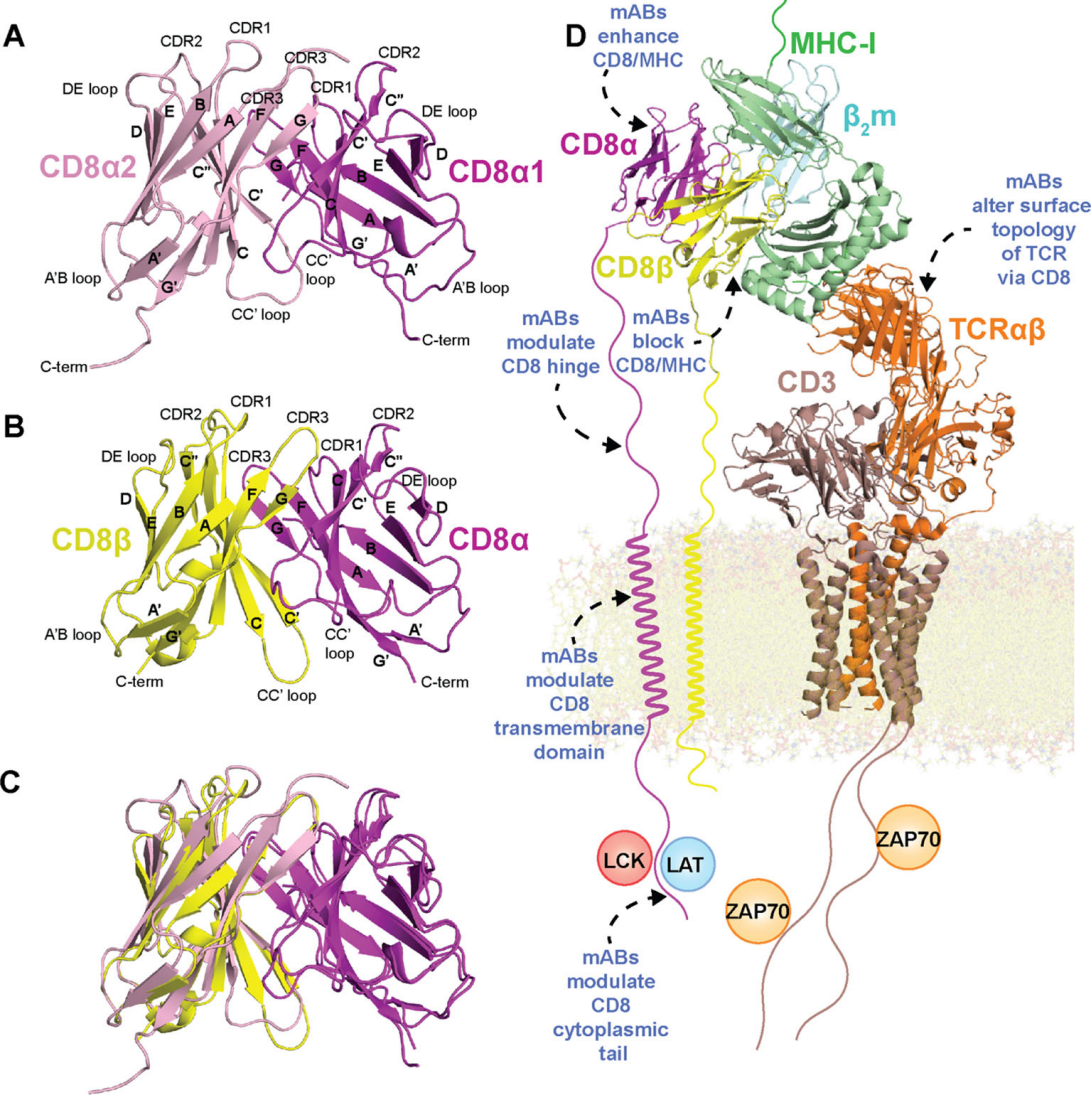
Structure of CD8 isoforms and placement within the immunological synapse with potential strategies for immunomodulation. **(A)** Crystal structure of mouse CD8αα ectodomain (PDB ID 1BQH). **(B)** Crystal structure of mouse CD8αβ ectodomain (PDB ID 2ATP). **(C)** Overlay of mouse CD8αα with mouse CD8αβ ectodomain. **(D)** Molecular model of the immunological synapse derived from the cryo-EM structure of the MHC-I/TCR/CD3 complex (PDB ID 7PHR) aligned to mouse CD8αα/H-2K^b^ (PDB ID 1BQH). The complex was adapted from a model presented by Pandey et al. (56). MHC-I heavy chain is colored green, β2m light chain colored cyan, peptide antigen colored salmon, CD8α colored magenta, CD8β colored yellow, TCR chains colored orange, and CD3 chains colored brown. Potential mechanisms for immunomodulation of CD8 structure/interactions by mABs (described in detail in the text) are highlighted with blue text/dotted arrows.

As new studies elucidate the complexities of immune regulation, there is a need to unravel how the CD8 co-receptor contributes to immune responses, either as a co-stimulator or co-repressor. This exploration naturally extends to the realm of monoclonal antibodies (mABs), potent immunomodulators known for their specificity and affinity, used in treating cancer, pathogen infections, autoimmune diseases, and for organ transplantation (29–31). Several anti-CD8 mABs have been discovered to exhibit interesting effects on immune cell signaling and development, highlighting CD8 and the immunological synapse as a novel strategy for targeted immunomodulation (**Tables 1**, **2**, **Figure 1D**). In this review, we highlight the structure and function of CD8 through the lens of its use as a target for immunomodulation. We also outline what is known (and what remains poorly understood) regarding the mechanistic understanding of how mABs engage with CD8 to promote or abrogate T cell signaling.

**Table 1:** Summary of CD8 structures available in the Protein Data Bank (PDB).

CD8 isoform	PDB ID	Other protein(s)	Reference
mouse CD8αβ	2ATP	–	(32)
human CD8α	1CD8	–	(33)
rhesus macaque CD8αα	2Q3A	–	(34)
bovine CD8αα	5EBG	–	(35)
swine CD8αα	5EDX	–	(35)
chicken CD8αα	5EB9	–	(35)
catshark CD8α	8HXS	–	(36)
human CD8αα	1AKJ	HLA-A*02:01	(37)
human CD8αα	3QZW	HLA-A*24:02	(38)
mouse CD8αα	1BQH	H-2K^b^	(39)
mouse CD8αβ	3DMM	H-2D^d^	(40)
chicken CD8αα	6LHG	BF2*04:01	(41)
chicken CD8αα	6LHF	BF2*15:01	(41)
mouse CDαα	1NEZ	TL	(42)
human CD8αα	7UMG	MR1	(43)
human CDαα C33A, S53N mutant	2HP4	–	(44)
mouse CDαα	2ARJ	YTS 105.18 antibody	(45)
mouse CD8αβ	3B9K	YTS 156.7 antibody	(46)
human CD8αα	7UVF	ZED8 antibody	(47)
human CD8α	8EW6	VHH5v2 antibody	(48)
human CD8α cytoplasmic tail fragment	1Q69	Lck fragment	(25)

**Table 2:** Summary of anti-CD8 monoclonal antibodies.

Target	Antibody	Immunomodulatory effect on CD8αβ T cell activation*	Reference(s)
CD8α	YTS 105.18	blocking	(45)
CD8β	YTS 156.7	blocking	(46)
CD8α	ZED8	neutral	(47)
CD8α	VHH5v2	blocking	(48)
CD8α	SK1	blocking	(49)
CD8α	DK25	blocking	(50, 51)
CD8α	3B5	blocking	(51)
CD8α	MCD8	neutral or limited blocking	(50, 51)
CD8α	CT-CD8a	block	(50–52)
CD8β	CT-CD8b	enhance	(50, 51)
CD8α	YTS 169.4	block	(53)
CD8β	53.5.8	block	(10)
CD8α	2ST8.5H7	block	(50)
CD8α	53.6.7	enhance	(50)
CD8α	OKT8	enhance	(50, 51)
CD8α	MRC OX-8	block	(54)
CD8β	KT112	enhance	(50, 55)
CD8α	32/M4	neutral	(50)
CD8α	C8/144B	neutral	(50)
CD8α	KT15	blocking	(52, 55)
CD8α	H59.101	blocking	(52)

*blocking/enhancing activity may differ by T cell subset due to different mechanisms for T cells expressing CD8αα, CD8αβ, or CD8ββ isoforms on their surface (see **Figures 7**, **8**).

## 2 CD8 structure and function

### 2.1 Distinct functional roles for CD8αα and CD8αβ in CD8^+^ T cell signaling and T cell development

CD8^+^ T cells play diverse roles in biology, ranging from antigen recognition in adaptive and innate immune responses for combating pathogen infection, cancer, and autoimmune disease, to cross-talking with B cell responses (57–59). Landmark studies revealed that the elimination of T cells expressing CD8α and CD8β chains resulted in a complete loss of immune cell-mediated cytotoxic responses (60–62). CD8 is expressed on CD8^+^ T cell surfaces in three possible isoforms: a CD8αα homodimer, a CD8ββ homodimer, and a CD8αβ heterodimer (63–65). Most commonly, one of the three isoforms is expressed in the absence of the other two. However, not all T cells express CD8; CD8^-^/CD4^+^ T cell and CD8^-^/CD4^-^ T cell subsets also exist (66). CD8^+^ T cell subtypes expressing CD8αα, CD8αβ, or CD8ββ are largely distinct (64, 67). CD8^+^ T cell subsets rarely express both CD8αα and CD8αβ (63, 64). CD8αβ is primarily expressed on the surface of conventional naïve cytotoxic T cells, mature cytotoxic T cells, memory T cells, natural killer T cells, mucosal associated invariant T cells, and γδ T cells (64, 67, 68). CD8αα is primarily expressed on the surface of intraepithelial T cells, thymocytes, conventional cytotoxic T cells, γδ T cells, memory T cells, natural killer T cells, mucosal associated invariant T cells, and dendritic cells (64, 69–72). CD8αα and CD8αβ are structurally similar but functionally distinct (72) (discussed in sections 2.2 through 2.6). The biological relevance of CD8ββ is largely contested since the CD8β chain is retained intracellularly in the absence of CD8α in some species but not others (65). Although the expression of CD8ββ has been demonstrated in some cell types, its structure/function remain poorly understood and are yet to be characterized (73).

Several reports from Sherman, Littman, and Mescher revealed CD8 as a main player in T cell activation where CD8/MHC-I binding serves as a TCR-activated adhesion-signaling system through cooperation with several other adhesion interactions (LFA-1/ICAM, VLA-ECM) (74–76). The adhesion property of CD8 with MHC-I was confirmed through several studies to be mediated by the MHC-I α3 domain (77–80). Purified MHC-I molecules immobilized on plastic were both necessary and sufficient to stimulate cytotoxic T cells in a TCR and CD8 dependent fashion (81, 82). Soluble anti-CD8 antibodies were able to inhibit TCR-activated binding and response to non-antigenic MHC-I (promoted by an anti-TCR antibody), further supporting a direct MHC-I/CD8 interaction contributes to T cell responses *via* an adhesion model (83). CD8αβ, and potentially CD8ββ, plays a role as a co-stimulatory molecule of CD8^+^ T cell signaling through its interaction with classical and non-classical MHC-I molecules (84) (discussed in section 2.3). CD8αα also interacts with classical and non-classical MHC-I molecules to function as a co-repressor for killer cell immunoglobulin-like receptors (KIRs) on natural killer (NK) cells (85). Both CD8αα and CD8αβ can associate with Lck via the CD8α cytoplasmic tail to promote CD3 ITAM phosphorylation, although the efficiency of this process seems to vary between CD8 isoforms (discussed in section 2.6). For example, in conventional CD8^+^ T cell subsets interacting with classical MHC-I molecules, CD8αβ has been observed to be ~100 times stronger of a co‐stimulator than CD8αα due to increased localization in lipid rafts for efficient Lck recruitment (86). Apart from its role as a co-stimulatory molecule of immune cell signaling, CD8αβ is involved in CD8^+^ T-lineage cell development, including thymocyte selection, maturation, and differentiation into memory and other classical and non-classical subsets (87–89). CD8αα is not a functional homolog of CD8αβ (72). While CD8αα’s function remains somewhat elusive, it has been associated with negative regulation of intestinal intraepithelial T cells that carry out cytotoxic functions in gastrointestinal and reproductive tracts (90). In this context, unlike CD8αβ, CD8αα decreases sensitivity of TCRs towards antigens (8). Additionally, CD8αα plays critical roles in generation of virus-specific memory T cells after infection (91, 92). Altogether, these studies reveal unique functional roles for CD8αα and CD8αβ isoforms in immune cell function and development, highlighting a wide range of responses to target for immunomodulation.

### 2.2 Evolutionary conservation of CD8α and CD8β chains

**Figure 2:**
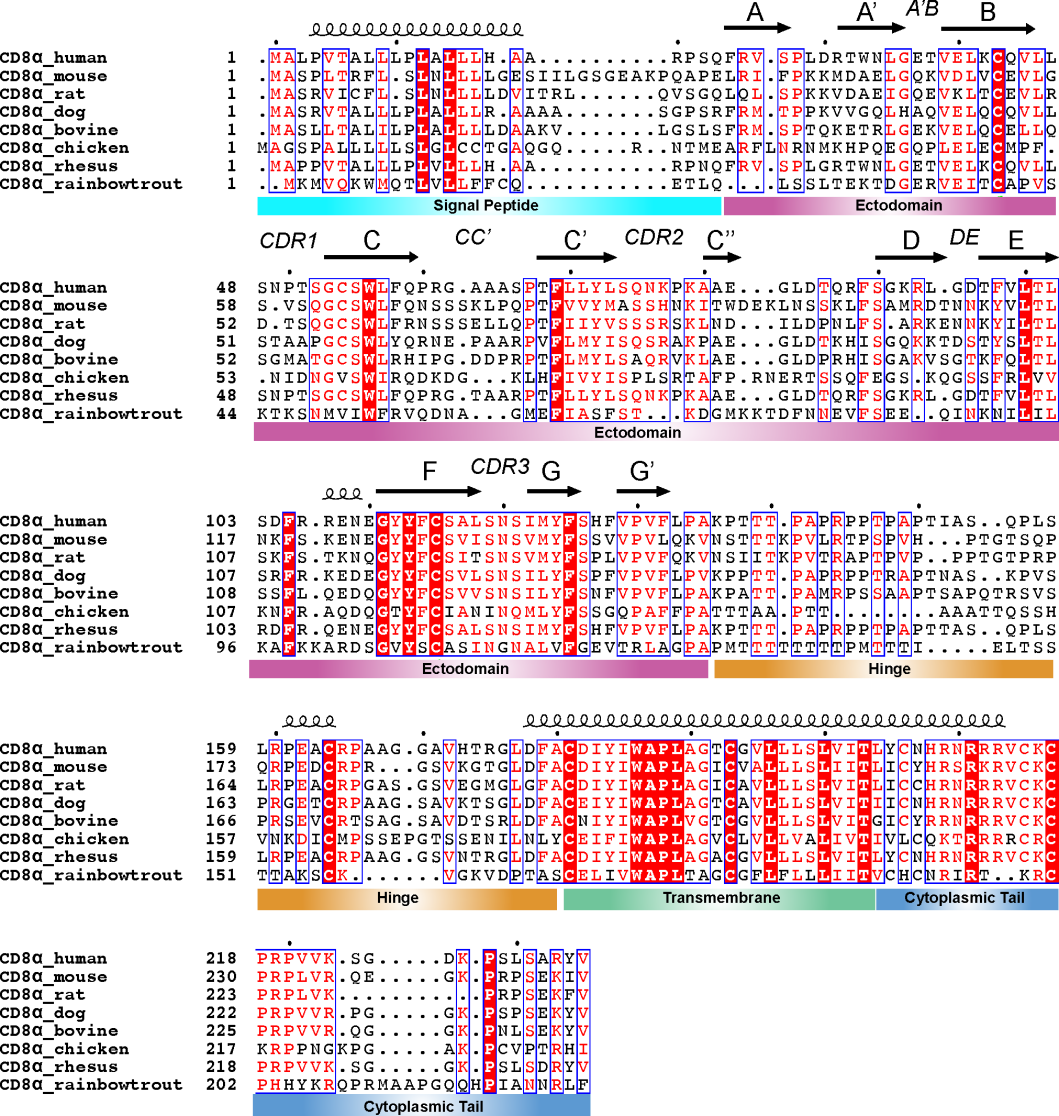
Sequence conservation of CD8α domains across species. Clustal Omega (v1.2.4) alignment (93) of sequences for CD8α from human (UniProt #P01732), mouse (UniProt #P01731), rat (UniProt #P07725), dog (UniProt #P33706), bovine (UniProt #P31783), chicken (UniProt #A0A8V0YYR0), rhesus monkey (UniProt #F7DXK3), and rainbow trout (UniProt #Q9IAL5). The secondary structure of each domain above the sequence is derived from the AlphaFold2 prediction of human CD8α (UniProt AF-P01732-F1). The alignment was processed in ESPript (v3) with domain disambiguation added manually (94). The dots above the sequence highlight the first residue (residue 1) and then every 10 residues after that using the first sequence as a reference.

**Figure 3:**
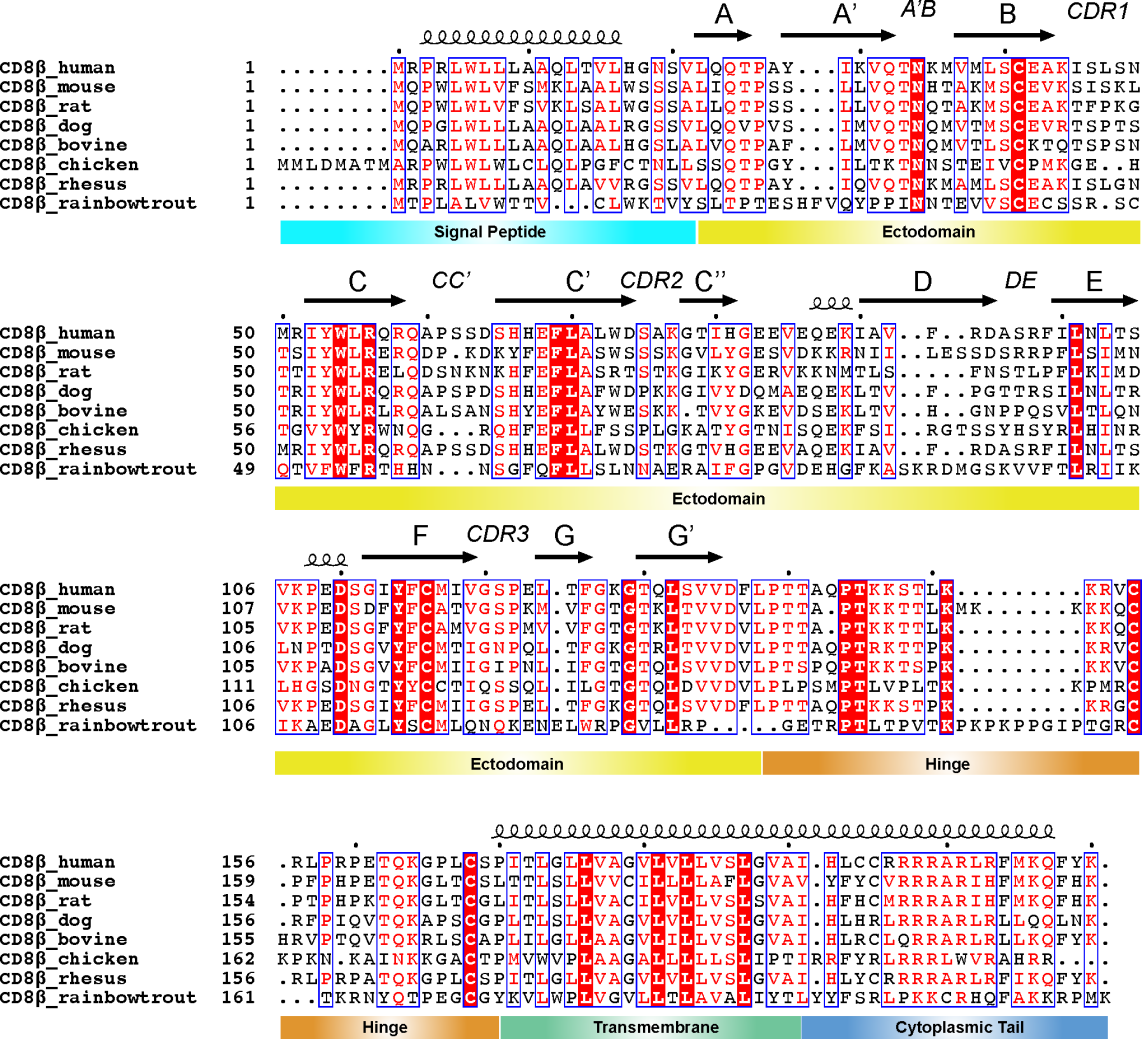
Sequence conservation of CD8β domains across species. Clustal Omega (v1.2.4) alignment (93) of sequences for CD8β from human (UniProt #P10966), mouse (UniProt #P10300), rat (UniProt #P05541), dog (UniProt #A0A8C0MNN1), bovine (UniProt #A7YW30), chicken (UniProt #A0A8V0Z0D5), rhesus monkey (UniProt #F7EXY4), and rainbow trout (UniProt #A0A8C7PPI8). The secondary structure of each domain above the sequence is derived from the AlphaFold2 prediction of human CD8β (UniProt AF-P10966-F1). The alignment was processed in ESPript (v3) with domain disambiguation added manually (94). The dots above the sequence highlight the first residue (residue 1) and then every 10 residues after that using the first sequence as a reference.

Amino acid sequence alignments of CD8α and CD8β chains across species indicate that despite sharing low sequence identity (~20 to 60% between species), several conserved sites are found in each of the ectodomain, hinge, transmembrane, and cytoplasmic tail (35, 40) (**Figures 2**, **3**). There are 24 and 21 fully conserved residues in CD8α and CD8β chains, respectively. The presence of these conserved sites suggests important structural and functional roles. For example, the ectodomain alignment highlights several conserved residues required for the stability of CD8’s immunoglobulin (Ig) fold and the intermolecular interface. The Cys residues in the ectodomain involved in the formation of an intramolecular disulfide bond, which stabilize CD8α and CD8β Ig-like folds, is also conserved. Cys residues in the hinge and transmembrane domains involved in an intermolecular disulfide bond, which stabilize CD8αα and CD8αβ dimers, are also conserved. Additionally, several CD8 residues involved in the interaction with MHC-I (ectodomain) and Lck (cytoplasmic tail) are partially conserved. Finally, several CD8 residues involved in transmembrane domain localization and interactions with lipid membrane are conserved. These conserved sites are attractive targets for immunomodulatory mABs that exhibit cross-species reactivity (discussed in section 3 and 4) (**Figure 1D**).

### 2.3 CD8 structure – the ectodomain binds to MHC-I and MHC-I related molecules to promote stability of MHC/TCR complexes

The CD8 ectodomain is an extracellular domain (amino acids 22-135 for CD8α and 22-138 for CD8β in humans) that takes on at least two forms: the CD8αα homodimer or CD8αβ heterodimer (7, 39). CD8ββ may provide a third isoform in some species, although its structure and function is poorly understood (65, 95). Crystal structures of the N-terminal globular domain of CD8αβ and CD8αα ectodomains reveal strikingly similar structural characteristics: an Ig-like fold consisting of eleven β-strands (termed A, A’, B, C, C’, C’’, D, E, F, G, and G’’) and six major loops (termed CDR1, CDR2, CDR3, DE, CC’, and A’B) (32) (**Figures 2**, **3**, **1A, B**). The dimer interface surface area differs between CD8αβ and CD8αα (~1914 Å^2^ versus ~2290 Å^2^) (32). The C_α_ RMSD between CD8αβ and CD8αα is ~1.16 Å, highlighting the overall similarity in the orientation of each CD8 chains in the two dimers (32) (**Figure 1C**).

**Figure 4:**
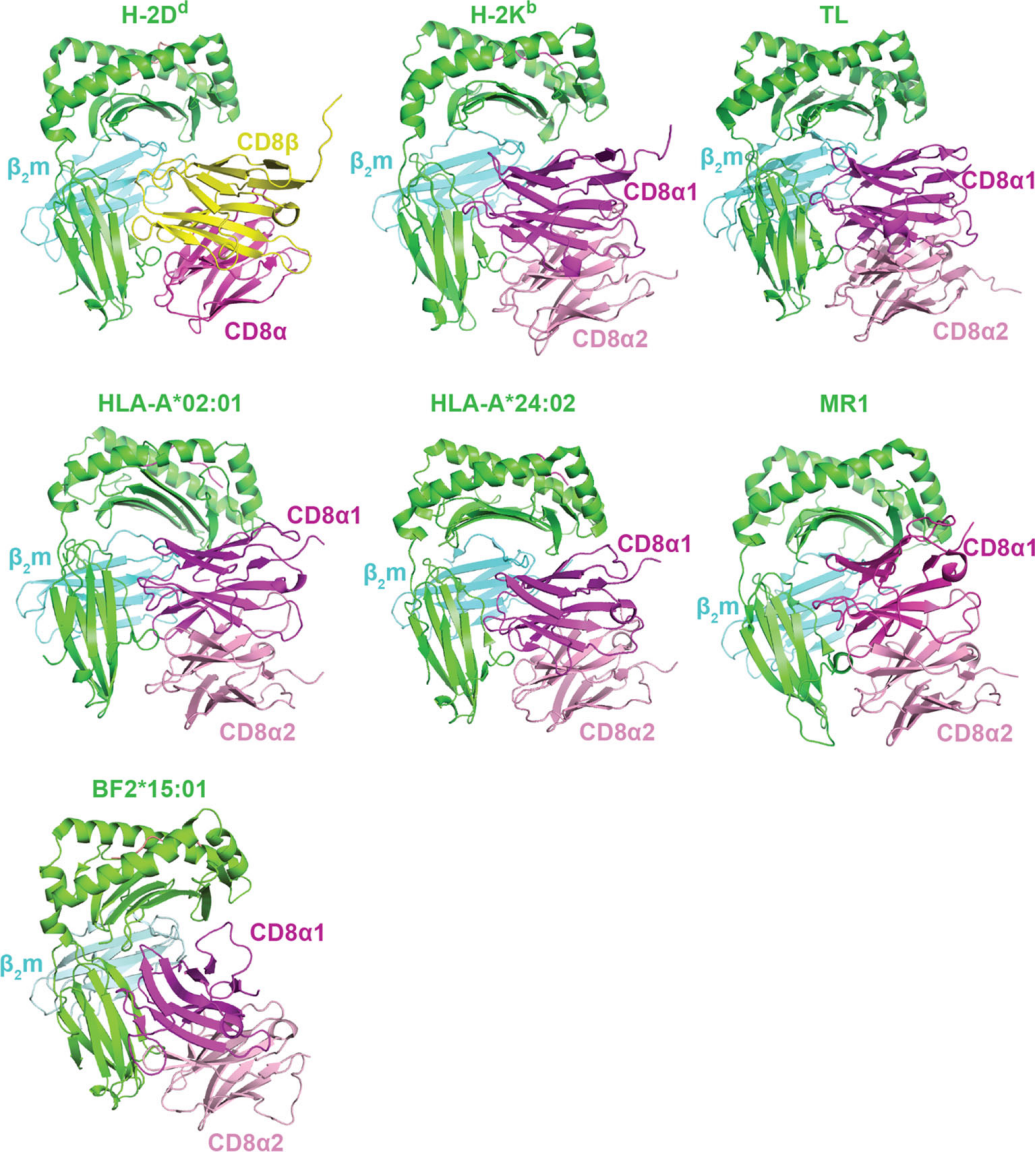
Binding modes of CD8αα and CD8αβ isoforms with classical and non-classical MHC-I molecules. A summary of crystal structures of CD8 ectodomain/MHC-I complexes. From top left to bottom right: mouse CD8αβ/H-2D^d^ (PDB ID 3DMM), mouse CD8αα/H-2K^b^ (PDB ID 1BQH), mouse CDαα/TL (PDB ID 1NEZ), human CD8αα/HLA-A*02:01 (PDB ID 1AKJ), human CD8αα/HLA-A*24:02 (PDB ID 3QZW), human CD8αα/MR1 (PDB ID 7UMG), and chicken CD8αα/BF2*15:01 (PDB ID 6LHF). MHC heavy chains are colored green, β2m light chains colored cyan, antigens colored salmon, CD8α chains are colored magenta/light pink, and CD8β chains are colored yellow.

CD8αα and CD8αβ ectodomains each interact with classical and non-classical MHC-I molecules using mostly similar binding modes (**Table 1**, **Figure 4**). While the exact positioning of the CD8 ectodomain varies across the different complexes, overall, the CD8β chain occupies a position equivalent to CD8α1, proximal to the T cell membrane, while the CD8α subunit of CD8αβ is positioned similarly to CD8α2, distal to the T cell membrane (**Figures 1D**, **4**). In crystal structures of mouse CDαβ/H-2D^d^ and mouse CD8αα/H-2K^b^ complexes, hydrogen bonds, salt bridges, and hydrophobic interactions stabilize interactions between CD8β and CD8α CDR1, CDR2, and CDR3 loops with a conformationally flexible loop on the MHC-I heavy chain α3 domain (39, 40) (**Figure 4**). Likewise, in crystal structures of human CDαα/HLA-A∗02:01 and human CD8αα/HLA-A∗24:02 complexes, CD8α contacts not only the MHC-I α3 domain, but also the MHC-I α2 helix and invariant light chain β2-microglobulin (β2m) (37, 38) (**Figure 4**). The structures of CDαα or CD8αβ with other HLA alleles (*i.e.*, HLA-B, HLA-C, HLA-G, etc.) are not available, but are expected to exhibit similar binding modes. Species-dependent differences in CD8/MHC binding modes have been reported. For example, crystal structures of chicken CDαα with BF2*04:01 and BF2*15:01 reveals two distinct modes: a classical antibody-like binding mode characteristic of most CD8/MHC-I complexes and a “face-to-face” binding mode that tilts the orientation of CD8αα homodimer relative to the MHC-I (41) (**Figure 4**), although the functional relevance of the second binding mode is unclear. The utility of anti-CD8 mABs for targeting CD8 ectodomain/MHC-I interactions is discussed in detail in section 4.

Classical MHC-I molecules are encoded by one of the most polymorphic genes described to date, with thousands of alleles that vary from person to person (96). In contrast, the gene encoding CD8 in humans is invariant and non-polymorphic, typically showing little variations between individuals, although limited allelic variation of CD8 is found in some species (97). Like CD8, non-classical MHC-I molecules generally exhibit minimal genetic diversity and hence have the potential to be therapeutic targets that are more universal compared to classical MHC-I (98). Mutations and polymorphisms in the MHC-I α3 domain have been shown to either enhance or disrupt CD8/MHC-I binding (80, 99). Mutations at conserved residues within the MHC-I α3 domain (*i.e.*, residues 222, 223, 227, 228, 229, 245) abrogate binding to CD8 and reduces T cell activation despite maintaining TCR binding (11, 37, 78, 100–103). For example, the well described MHC-I α3 domain D227K/T228A mutant disrupts association with CD8 by eliminating a hydrogen bond between D227 (on MHC-I) and Y51 (on CD8α) as well as contacts between T228A (on MHC-I) and T30/N99 (on CD8α) (11, 37, 40).

Surface plasmon resonance (SPR) and micropipette adhesion frequency assay have been used to quantify differences between CD8αα and CD8αβ ectodomains interactions with classical or non-classical MHC-I molecules in solution (i.e., three-dimensional, or 3D, binding) and on T cell membrane (i.e., two-dimensional, or 2D, binding). The formation of CD8/MHC-I complexes doesn’t seem to be largely dependent on the identity of the bound peptide antigen for most cases (104, 105). CD8αα and CD8αβ usually exhibit similar affinities to the same MHC-I molecule (40, 105), which is interesting considering functional differences between the two CD8 isoforms. In general, CD8αα and CD8αβ interact with classical and non-classical MHC-I molecules with moderate to weak affinities in the range of K_D_ ~10 to 500 µM in 3D (40, 42, 43, 103, 106) and A_c_K_A_ ~10^-6^ μm^4^ in 2D (12, 16). Most mABs have a 3D K_D_ ~ nM and 2D A_c_K_A_ ~10^-1^ μm^4^ for their specific antigens (107), which should have no problem blocking CD8/MHC-I interactions. Some alleles, such as HLA-A*68:01, HLA-B*48:01, and HLA-E bind CD8αα extremely weakly (K_D_ ≥ 1 mM), which is biologically defined as non-binding (40, 42, 43, 103, 106). The weaker affinities of CD8 for the latter group of MHC-I molecules result from either *i)* polymorphisms of residues within the MHC-I α3 domain directly influences interactions with CD8α and CD8β, or *ii)* alternations in the conformation of the α3 domain (103). CD8 also plays a crucial role in modulating CD8^+^ T cell activation through non-classical MHC-I molecules, such as MHC-Ib molecules (TL, H2-Q10, H2-T22, Qa-1b), small molecule metabolite antigen presenting MR1, and lipid antigen presenting CD1 (42, 43, 108–110). CD8 seems to also function as a co-stimulatory molecule for conventional CD8^+^ T cells, γδ T cells, natural killer cells, and MAIT cells via interactions with MR1 (43). However, as shown for classical MHC-I/TCR interactions, high affinity small molecule antigen/MR1/TCR interactions do not strictly require CD8 engagement, while immune cell responses to low affinity MR1/TCR interactions are reduced or abrogated in the absence of CD8 (43). CD8αα homodimers and CD8αβ heterodimers bind MR1 in a manner similar to classical MHC-I (43) (**Figure 4**). Unlike with most MHC-I molecules, TL shows a stronger affinity to CD8αα compared to CD8αβ (K_D_ = 12 μM for CD8αα versus > 90 μM for CD8αβ) (111). This disparity is not a result of a different binding mode relative to classical MHC-I; instead, the enhanced CD8αα affinity is attributed to the formation of additional hydrogen bonds (111). Overall, these structural and biophysical data shed light on the specific molecular interactions governing the binding of CD8αα or CD8αβ to classical and non-classical MHC-I molecules, elucidating unique features of their complex formation, stability, and cellular function.

Dynamics and conformational heterogeneity is also an intrinsic feature of the MHC-I that regulates its function (112, 113). All-atom molecular dynamics simulations have hinted at the MHC-I α3 domain’s ability to sample a wide range of conformations, especially in the absence of bound peptide (114–118). A global analysis of B-factors, an implicit metric to identify the flexibility of atoms, for X-ray crystal structures of human MHC complexes reveals the MHC-I α3 domain as a region with significant dynamic properties (112). The MHC-I α1 and α2 domains (i.e., antigen binding groove) also display conformational plasticity, which may be allosterically coupled to the α3 domain (112). Gao et al. have suggested that differences in affinities of classical and non-classical class I MHC molecules for CD8 can be attributed to conformational changes in the α3 domain (residues 223-229) (103). Solution NMR (119–121) and hydrogen/deuteration exchange mass spectrometry (122, 123) measurements of MHC-I molecules have also experimentally confirmed the α1, α2, and α3 domains are conformationally labile in both empty and peptide-bound states in an allele dependent manner. Since peptides modulate stability and conformational plasticity of the MHC-I (116, 121, 123), MHC-I/CD8 interactions might display a peptide-dependence. Evidence against this comes from biophysical measurements of HLAs with different peptides, which reveals very similar affinities with CD8 (11). However, this hypothesis is supported by findings that empty HLA-B molecules, which are known to be conformationally diverse in structure, show stronger CD8 binding affinity than those loaded with specific peptides, potentially due to an enhanced ability to sample optimal α3 domain conformations for binding (114, 119, 124). Together, these results suggest that MHC-I/CD8 affinities are similar for high affinity peptides that stabilize the MHC-I, while CD8 could have enhanced affinity for empty MHC-I and MHC-I bound to low affinity peptides that do not stabilize the MHC-I. Finally, mutations/polymorphisms in the MHC-I α3 domain likely alter its conformation landscape to influence, either positivity or negatively, interaction with CD8 to influence immunological outcomes (42, 80, 103, 116, 117, 120, 125, 126).

### 2.4 CD8 structure – the hinge contributes to the co-regulator function and relay of signaling

The CD8 hinge region (amino acids 136-182 for CD8α and 139-170 for CD8β in humans) connects the N-terminal globular domain with the membrane-embedded transmembrane domain. The CD8 hinge (also called the “stalk”) is thought to play key roles in communication between the transmembrane and ectodomains, ultimately influencing CD8/MHC-I binding and relaying of signals to CD3 ITAMS via Lck (53, 54, 127). Both CD8α and CD8β hinge sequences are rich in proline, threonine, and serine amino acids, but differ in primary sequence, physical length, and glycosylation patterns (53, 128) (**Figures 2**, **3**). Several CD8/MHC-I crystal structures contained CD8 hinge regions in the expression construct, but electron density for those atoms are missing, suggesting the hinge is unstructured and/or conformationally dynamic (40). In agreement with this, solution NMR experiments suggest that the CD8α hinge lacks a well-ordered structure, is intrinsically flexible, and can undergo cis-trans proline isomerization to sample different functionally relevant states (127). The CD8β hinge contains fewer prolines residues, which likely restricts its conformational landscape relative to CD8α hinge. The CD8 hinge undergoes O-linked glycosylation with sialic acid, which likely influences its structure and function (128–131). For example, immature CD8^+^ thymocytes exhibit different levels of CD8 hinge O-linked glycosylation relative to mature CD8^+^ T cells, altering the affinity of CD8 for MHC-I for different CD8^+^ immune cell subsets (129, 132). Modulating CD8 hinge glycosylation could influence association or orientation of CD8αα or CD8αβ ectodomains with classical and non-classical MHC-I molecules by controlling the hinge’s contributions to “cis” or “trans” binding modes (133). One possible strategy for immunomodulation here is altering the glycosylation pattern of the CD8 hinge using sialidase enzymes, a strategy that has been shown to modulate immune cell activation (134).

### 2.5 CD8 structure – the transmembrane domain traffics CD8 to the cell surface and promotes CD8 dimerization

The CD8 transmembrane domain (amino acids 183-203 for CD8α and 171-191 for CD8β in humans) plays key roles in intracellular trafficking to the cell surface and assembly of CD8 homo- and heterodimers (135, 136). The CD8α and CD8β transmembrane domains each contain a membrane-proximal Cys residue that forms an intermolecular disulfide bond to stabilize CD8αα homodimers and CD8αβ heterodimers (135). While no atomic structures are currently available for the CD8α and CD8β transmembrane domains, they are predicted by as type I single-pass integral membrane sequences with α-helical secondary structures (5).

### 2.6 CD8 structure – the cytoplasmic tail mediates localization into signaling lipid rafts and recruits Lck to promote TCR signaling

**Figure 5:**
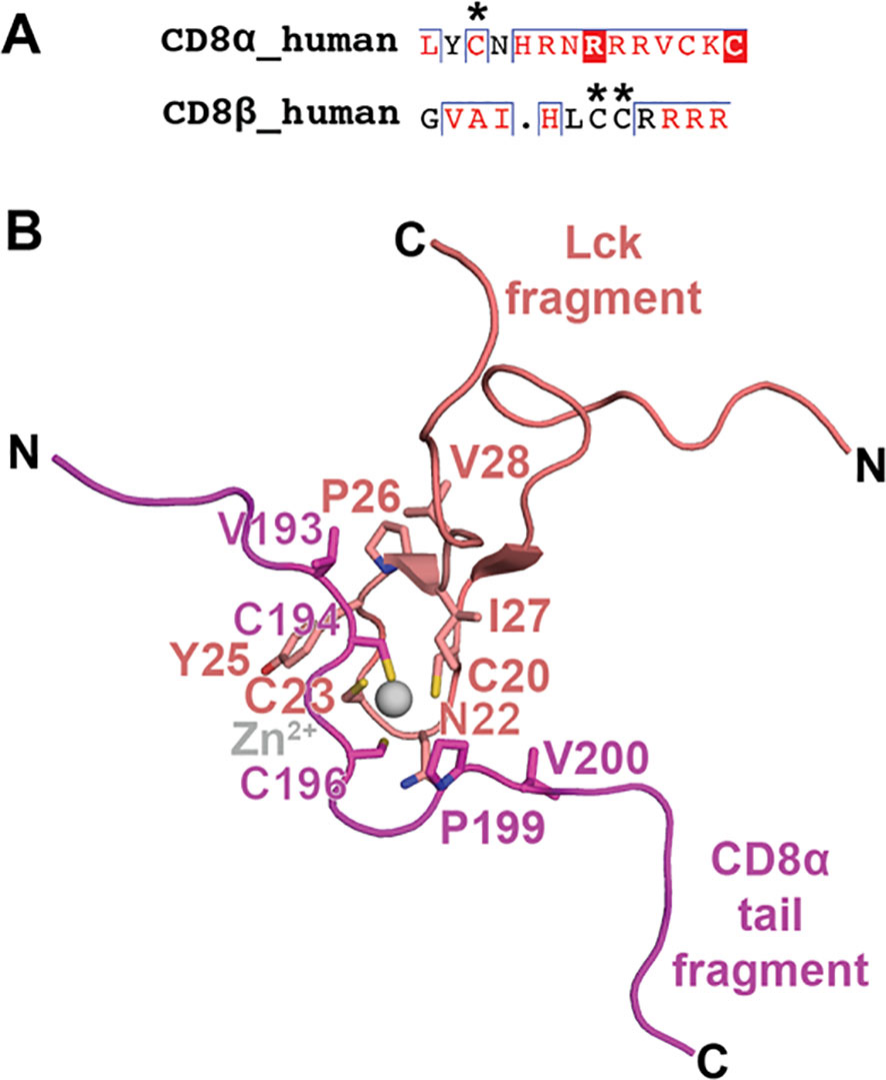
CD8α and CD8β cytoplasmic tails: palmitoylation and binding mode to Lck. **(A)** A sequence alignment of human CD8α and human CD8β cytoplasmic tails is shown with palmitoylated residues indicated with an asterisk. **(B)** NMR structure of the interaction between a fragment of the cytoplasmic tail from CD8α with a fragment from Lck (PDB ID 1Q69). The interaction is coordination by a Zn^2+^ ion and Cys residues on CD8α and Lck. Other residues participating in the interaction are also shown as sticks. CD8α is colored magenta, Lck is colored salmon, and Zn^2+^ is colored gray.

The CD8 cytoplasmic tail (amino acids 204-235 for CD8α and 192-210 for CD8β in humans) plays several essential roles in CD8’s function as a co-regulator: *i)* it helps localize CD8 to lipids rafts containing membrane and *ii)* it recruits Lck and/or LAT to the immunological synapse to promote TCR signaling. As a result of these important features, the CD8 cytoplasmic tail also contributes to thymic selection and immune cell maturation (16, 137–140). Both CD8α and CD8β are palmitoylated (covalent addition of palmitic acid at Cys residues) in the cytoplasmic tail, which promotes the incorporation of CD8 into lipid rafts which are enriched in the immunological synapse (141–144). CD8β contains more palmitoylation sites than CD8α, potentially contributing to differences in the localization of CD8αα homodimers and CD8αβ heterodimers in lipid rafts to alter signaling efficiency (95, 143) (**Figure 5A**).

The cytoplasmic tail of CD8α, but not CD8β, contains a membrane-proximal CxC motif required for Lck binding (24, 145, 146). Thus, in principle, CD8αα and CD8αβ can each associate with two and one Lck molecules, respectively. While CD8β doesn’t contain a Lck binding site, CD8β seems to be required for association and activity of Lck with CD8α, potentially due to the role of its palmitoylation site and recruitment to the cell membrane (147, 148). A partial structure of the CD8α/Lck complex was determined by solution NMR (25) (**Figure 5B**). The structure suggests that the CD8α/Lck complex is stabilized by two mechanisms. First, a zinc ion is coordinated by the CxC motif of the CD8α cytoplasmic tail and Cys residues on Lck that adopt a zinc-hairpin structure (25, 149). Second, CD8α establishes hydrophobic interactions with Lck, notably through residues V193, P199, and V200, which further stabilize the complex by engaging with the Lck hairpin (25). The dissociation of Lck from the CD8α cytoplasmic tail is a complex regulatory event orchestrated by inhibitory receptors, such as LAG3 (150). Despite the absence of a CxC motif, the CD8β chain seems to be critical for CD8αβ co-receptor function (147, 148, 151, 152).

Whether CD8αα associates in an appreciable way with Lck is a contentious point in the field. It is clear that the co-receptor function of CD8αα and CD8αβ is quite different, which could be related to differences in interactions with MHC, Lck, or LAT (8, 72). However, a question remains on whether these differences are tied to changes in affinities of CD8αα and CD8αβ for Lck, or due to other contributing factors (*i.e.*, ability of CD8β to recruit proteins into kinase-rich lipid rafts for enhanced signaling (95, 143)). Several studies in hybridomas and thymocytes imply that Lck association with CD8α chain is reduced in cell lines expressing CD8αα compared with CD8αβ, resulting in abrogated T cell activation (147, 148, 151, 152). However, bimolecular fluorescence complementation assays suggest Lck has the ability to associate with both CD8αα and CD8αβ, which are each efficiently recruited to the immunological synapse through interactions with the MHC-I (86). The authors suggest CD8αβ’s ability to act as a more robust co-receptor than CD8αα is tied to the CD8β chain’s action in recruiting Lck in the appropriate lift raft environment rather than stark differences in intrinsic Lck affinity, MHC-I binding, or recruitment to the immunological synapse (86). Further study in cell lines associated with CD8αα expression and function could provide additional insights (90). The cytoplasmic tail of CD8α also binds to LAT (153). While structure insights into CD8/LAT interactions are lacking, the complex seems to be mediated by an overlapping epitope with Lck since Lck and LAT binding to CD8 is mutually exclusive (153).

Ultimately, TCR signaling may involve direct competition of Lck and LAT for the cytoplasmic tails of CD8 and CD4 co-receptors, which differ in their affinity for and occupancy of Lck (2, 153). Some reports suggest that only a small percentage of CD4 and CD8 coreceptors engage with Lck (6.8% CD4 vs 0.6% CD8αβ), such that TCRs are required to scan multiple coreceptor molecules to identify a Lck-coupled state for signaling (140). Other studies have measured a much higher Lck occupancy where CD4 is also reported to be higher than CD8αβ (~100% CD4 vs 60% CD8αβ) (154). The stark differences between measurements has been attributed to sample preparation, processing, or experimental conditions (154), although consistency is observed in increase of Lck occupancy by CD4 relative to CD8αβ. CD4/Lck and CD8/Lck occupancy may also vary depending on specific cell type or signaling conditions (154, 155). It has been suggested that the increased occupancy of Lck by CD4 relative to CD8αβ compensates for low affinity of CD4/MHC-II assemblies (154).

### 2.7 CD8 features used for CAR T cell engineering

Unlike conventional CD8^+^ T cells, chimeric antigen receptor (CAR) T cells contain CARs engineered to recognize target cell surface antigens, usually independently of MHC-I based antigen presentation (156). However, CARs are still designed with structural components to contain many properties important for conventional T cell signaling: a target recognition domain (typically an antibody, nanobody, or other ligand), a hinge region as a spacer, a transmembrane domain, a co-stimulatory domain (typically derived from CD28), and a cytoplasmic tail that serves as a signaling motif (typically derived from CD3) (156). CARs are often engineered to include CD8α’s hinge region, which regulates CAR T cell receptor flexibility, antigen recognition, and signaling (127, 157–159). Further engineering of CAR length and sequence composition can enhance the properties of the hinge even further. For example, removal of glycine residues in the CD8α hinge reduces the flexibility of second generation CARs, preventing overactivation of CAR T cells by altering steric hindrance and spatial accessibility CAR recognition domain for target antigens (158). Flanking the CD8 hinge, the CD8α transmembrane is also often incorporated into engineered CARs where it dictates receptor surface expression level and signaling activity (157, 160). Interestingly, CAR T cells engineered with CD8α hinge and transmembrane domains are less susceptible to activation-induced cell death compared to those derived from other receptors, such as CD28 (161). It would be interesting to evaluate functional properties of CD8 hinge and transmembrane domains (*i.e.*, cis-proline isomerization, palmitoylation, homodimerization) with respect to CAR T cell function.

## 3 Immunomodulatory CD8 mutations

Mutations in CD8, obtained through genetic mutation, naturally occurring polymorphisms, or experimental engineering, can lead to downregulation or upregulation in T cell signaling (44, 162–164). For example, familial missense mutations in CD8α cause CD8 deficiency due to protein misfolding (135, 138). The lack of CD8α expression in patients results in high percentages of CD8^–^ T cells and dysregulated immune responses (49, 162). In contrast, engineered CD8 mutations have been used to improve antigen sensitivity for low-affinity MHC-I/TCR complexes (163). Examples of engineered enhancing variants include S53N, S53G, and C33A/S53N for CD8α (located on the C strand and CDR2 loop) and S53L and L58R for CD8β (located on the CDR2 loop) (44, 162–164). The X-ray structure of the engineered human CDαα C33A/S53N mutant suggests that the enhanced affinity is due to a new electrostatic interaction between N53 of CD8α1 and D223 on the MHC-I α3 domain (44). Engineered CD8 molecules could be used enhance the sensitivity of antigen recognition for low-affinity TCRs (163).

## 4 CD8 interactions with monoclonal antibodies

Anti-CD8 mABs have seen widespread use in immunology research, immunotherapy, and modern medicine. They are common reagents used for flow cytometry to evaluate/isolate T cell subsets, in T cell stimulation assays, to elucidate TCR/CAR signaling mechanisms, and to characterize disease-specific antigen responses (50, 57, 165). Beyond their use in basic immunology research, anti-CD8 mABs show great promise as immunomodulatory therapeutics in medicine. For example, *in situ* and *in vivo* studies have suggested anti-CD8 mABs have the potential to block detrimental activity of autoreactive CD8^+^ T cells in autoimmune diseases, such as glomerulonephritis (166), type 1 diabetes (167, 168), and rheumatoid arthritis (169). Anti-CD8 antibody therapy also could play a role in mitigating transplant rejection related to HLA mismatch between patients (31, 170). In a completely separate application, treatment of CD8^+^ T cells with agonistic anti-CD8 mABs has been shown to enhance tumor targeting and killing by cytotoxic T cells (171). However, in some cases, mAB binding to CD8 is “neutral” and does not influence T cell activity (50). While these findings are very promising, there are caveats associated with the use of anti-CD8 mABS since it has been suggested that they alters the phenotype and behavior of CD8+ T cells, which have positive or negative effects to therapy outcome (172). The full utility of anti-CD8 mABs in therapy remains to be characterized, and requires further studies evaluating a panel of different mABs with different disease types.

Ultimately, the CD8/mAB binding sites and modes relative to the cell membranes dictate the mechanisms governing their immunomodulatory properties (**Figure 1D**). Given their wide range of binding modes and functional outcomes, it is essential to characterize how anti-CD8 mABs structurally engage with CD8αα and/or CD8αβ isoforms. Characterizing atomic structures of CD8/mAB complexes not only uncovers mechanistic insights into mAB function, but also provides a molecular blueprint to improve affinity, activity, and specificity. Several structures of CD8/mAB complexes have been reported in the literature (**Table 1**), however structural insights into many important CD8/mAB interactions remain lacking (**Table 2**). Antibodies targeted to the CD8α chain have the potential to interact with both CD8αα and CD8αβ isoforms, depending on the recognized epitope, while antibodies against the CD8β chain are specific for CD8αβ or CD8ββ (if present). Thus, defining binding epitopes and structures of CD8/mAB complexes and quantifying CD8/mAB binding affinities reveals important information on the CD8^+^ T cell subtypes with potential for immunomodulation. Finally, structural elucidation informs whether anti-CD8 mABs are expected to be restricted to certain species (*i.e.*, the mAB recognizes a non-conserved CD8 epitope) or exhibit cross-reactivity against many species (*i.e.*, the mAB recognizes a conserved CD8 epitope). The information known about how mABs bind to CD8 to carry out their function is discussed below.

### 4.1 YTS 105.18 antibody blocks CD8^+^ T cell signaling by two distinct mechanisms

**Figure 6:**
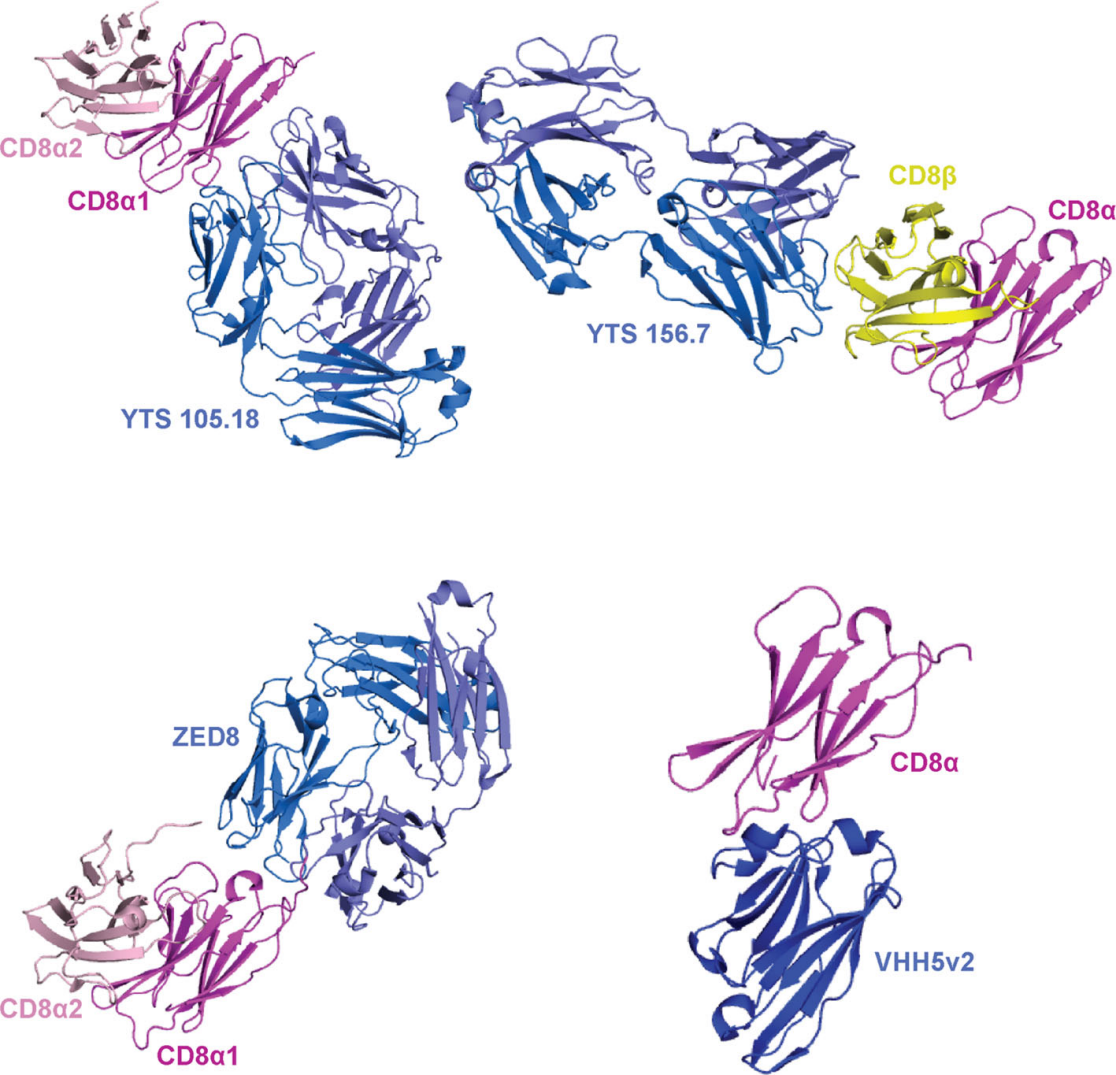
Binding modes of mABs to CD8αα and CD8αβ. A summary of crystal structures of CD8 ectodomain/mAB complexes. From top left to bottom right: mouse CDαα/YTS 105.18 (PDB ID 2ARJ), mouse CD8αβ/YTS 156.7 (PDB ID 3B9K), human CD8αα/ZED8 (PDB ID 7UVF), and human CD8α/VHH5v2 (PDB ID 8EW6). mABs are colored blue/light blue, CD8α chains are colored magenta/light pink, and CD8β chains are colored yellow. The orientation of the magenta CD8α is maintained throughout for comparison.

**Figure 7:**
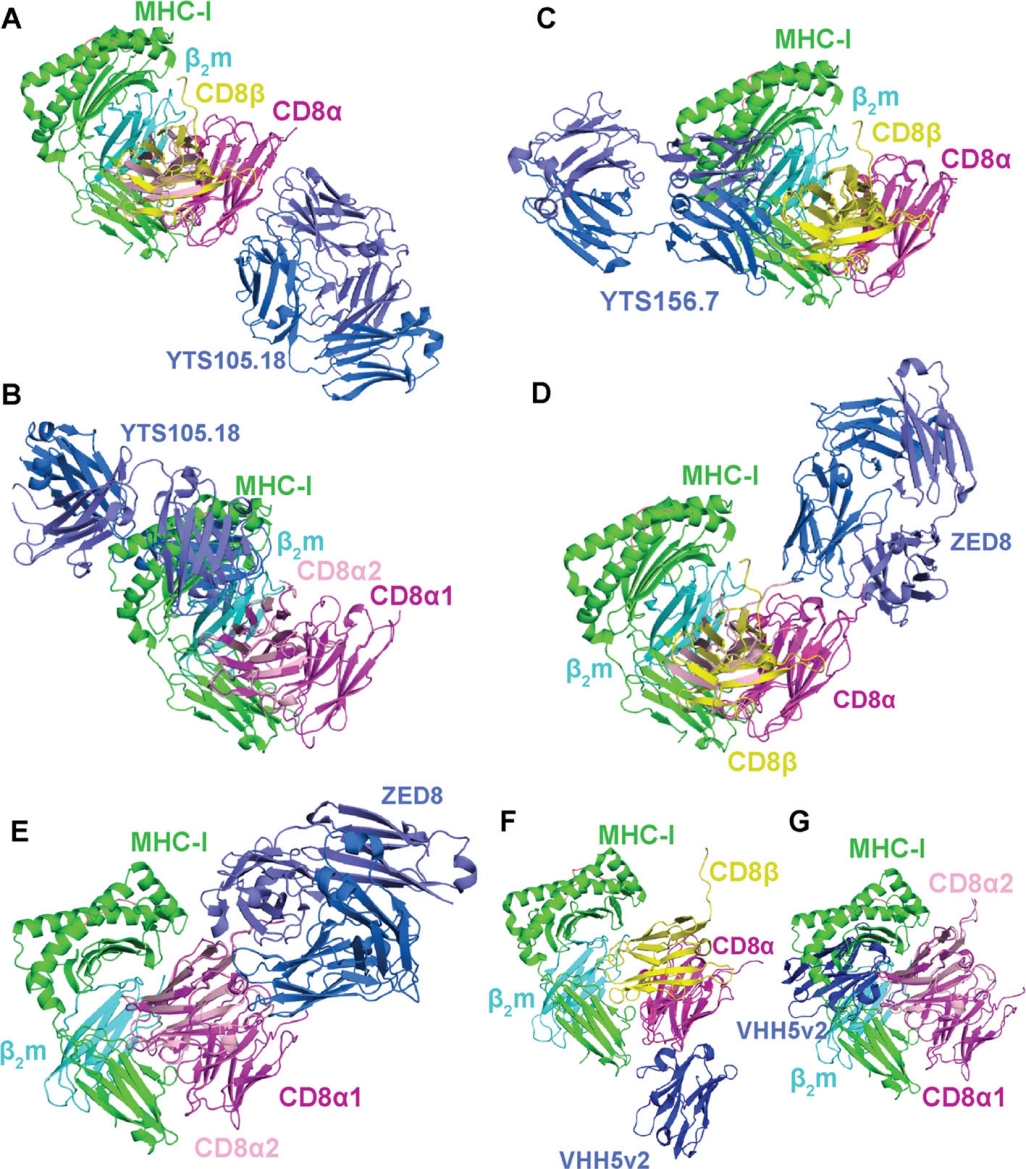
Potential for different mABs to block or enhance CD8αα and CD8αβ interactions with classical and non-classical MHC-I molecules. **(A)** Overlay of mouse CDαα/YTS 105.18 (PDB ID 2ARJ) with mouse CD8αβ/H-2D^d^ (PDB ID 3DMM). In this binding mode, no steric hindrance of CD8/MHC is seen. **(B)** Overlay of mouse CDαα/YTS 105.18 (PDB ID 2ARJ) with mouse CD8αα/H-2K^b^ (PDB ID 1BQH). In this binding mode, steric hindrance of CD8/MHC is seen. **(C)** Overlay of mouse CD8αβ/YTS 156.7 (PDB ID 3B9K) with mouse CD8αβ/H-2D^d^ (PDB ID 3DMM). In this binding mode, steric hindrance of CD8/MHC is seen. **(D)** Overlay of human CD8αα/ZED8 (PDB ID 7UVF) with mouse CD8αβ/H-2D^d^ (PDB ID 3DMM). In this binding mode, no steric hindrance of CD8/MHC is seen. **(E)** Overlay of human CD8αα/ZED8 (PDB ID 7UVF) with mouse CD8αα/H-2K^b^ (PDB ID 1BQH). In this binding mode, no steric hindrance of CD8/MHC is seen. **(F)** Overlay of human CD8α/VHH5v2 (PDB ID 8EW6) with mouse CD8αβ/H-2D^d^ (PDB ID 3DMM). In this binding mode, no steric hindrance of CD8/MHC is seen. **(G)** Overlay of human CD8α/VHH5v2 (PDB ID 8EW6) with mouse CD8αα/H-2K^b^ (PDB ID 1BQH). In this binding mode, steric hindrance of CD8/MHC is seen. In all panels, the orientation of the magenta CD8α1 is maintained throughout for comparison. MHC-I heavy chains are colored green, β2m light chains colored cyan, antigens colored salmon, CD8α chains colored magenta/light pink, CD8β chains colored yellow, and mABs colored blue/light blue.

YTS 105.18, a rat IgG2_a_ anti-CD8α monoclonal antibody, like most anti-CD8 mABs identified to date is widely used for the blocking of CD8^+^ T cell activation (173). YTS 105.18 can induce tolerance to xenograft transplantation and reduce insulin-dependent diabetes mellitus by downregulating CD8^+^ T cell activity (174, 175). YTS 105.18 has also been used to show that CD8^+^ T cell blockade plays a role in promoting the development of CD4^+^ regulatory T cells (176). Insights into the blocking mechanism of YTS 105.18 came from an X-ray structure of the mouse CD8αα/YTS 105.18 complex (45, 46) (**Figures 6**, **7A, B**). YTS 105.18 recognizes an epitope spanning the A, A′ and B strands of CD8α, suggesting it can recognize both CD8αα homodimers and CD8αβ heterodimers with distinct mechanisms. In a model where YTS 105.18 binds to CD8αβ, the blocking activity of YTS 105.18 is likely not mediated by sterically hindering CD8αβ/MHC interactions (**Figure 7A**), and could be due to disruptions of the topology of the interfacial membranes between the T cell and the MHC-expressing cell (e.g., immunological synapse) (45, 46) (**Figure 1D**). In contrast, for T cells expressing CD8αα on the surface, YTS 105.18 would have the potential to sterically hinder CD8αα/MHC-I interactions (**Figure 7B**).

### 4.2 YTS 156.7 antibody blocks CD8^+^ T cell signaling by inhibiting CD8/MHC-I binding

YTS 156.7 is a rat IgG2_b_ anti-CD8β monoclonal antibody which, like YTS 105.18, blocks CD8^+^ T cell activation. YTS 156.7 has been used to deplete CD8^+^ T cells *in vivo* to enable immunomodulatory applications similar to YTS 105.18 . Insights into the mechanism of YTS 156.7 were revealed by an X-ray structure of the mouse CD8αβ/YTS 156.7 complex (46) (**Figures 6**, **7C**). YTS 156.7 recognizes an epitope spanning the CDR1, C-C′ and D–E loops of CD8β (46), which is distinct from the CD8α epitope recognized by YTS 105.18. The blocking activity of YTS 156.7 for T cells expressing CD8αβ is most likely mediated by steric hinderance of CD8αβ/MHC-I interactions (46) (**Figure 7C**). Since YTS 156.7 is specific for the CD8β chain, it would likely not influence CD8^+^ T cells expressing CD8αα.

### 4.3 ZED8 and VHH5v2 antibodies used in immunoPET imaging of CD8

Immuno-positron emission tomography (immunoPET) is a technique used for molecular imaging of proteins by combining PET imaging with radioisotope-labeled mABs targeted to a protein of interest (177). ImmunoPET protocols utilizing anti-CD8α mABs, such as ^89^Zr-labeled ZED8 and ^18^F-labeled VHH5v2, have enabled imaging of CD8^+^ T cells in solid tumors and xenografts over time (47, 48). ImmunoPET anti-CD8 mABs may also have immunomodulating properties depending on their binding mechanisms. ZED8 antibody recognizes an epitope on CD8α spanning the A’B loop, the α-helix flanking the F strand, and the ectodomain/hinge transition, and does not appear to alter CD8^+^ T cell activation (47, 178). In line with this, the X-ray structure of human CD8αα with ZED8 antibody suggests it does not sterically hinder either CD8αβ/MHC-I or CD8αα/MHC-I interactions (47) (**Figures 7D, E**). The VHH5v2 antibody recognizes an epitope on CD8α spanning the CDR1 and DE loops with affinity of ~500 pM. The immunomodulatory properties of VHH5v2 have not been examined. However, the X-ray structure of human CD8α with VHH5v2 suggests it likely does not sterically hinder CD8αβ/MHC-I interactions but could disrupt CD8αα/MHC-I complex formation (48) (**Figures 7F, G**).

### 4.4 Immunomodulation of CD8 by other monoclonal antibodies

**Figure 8:**
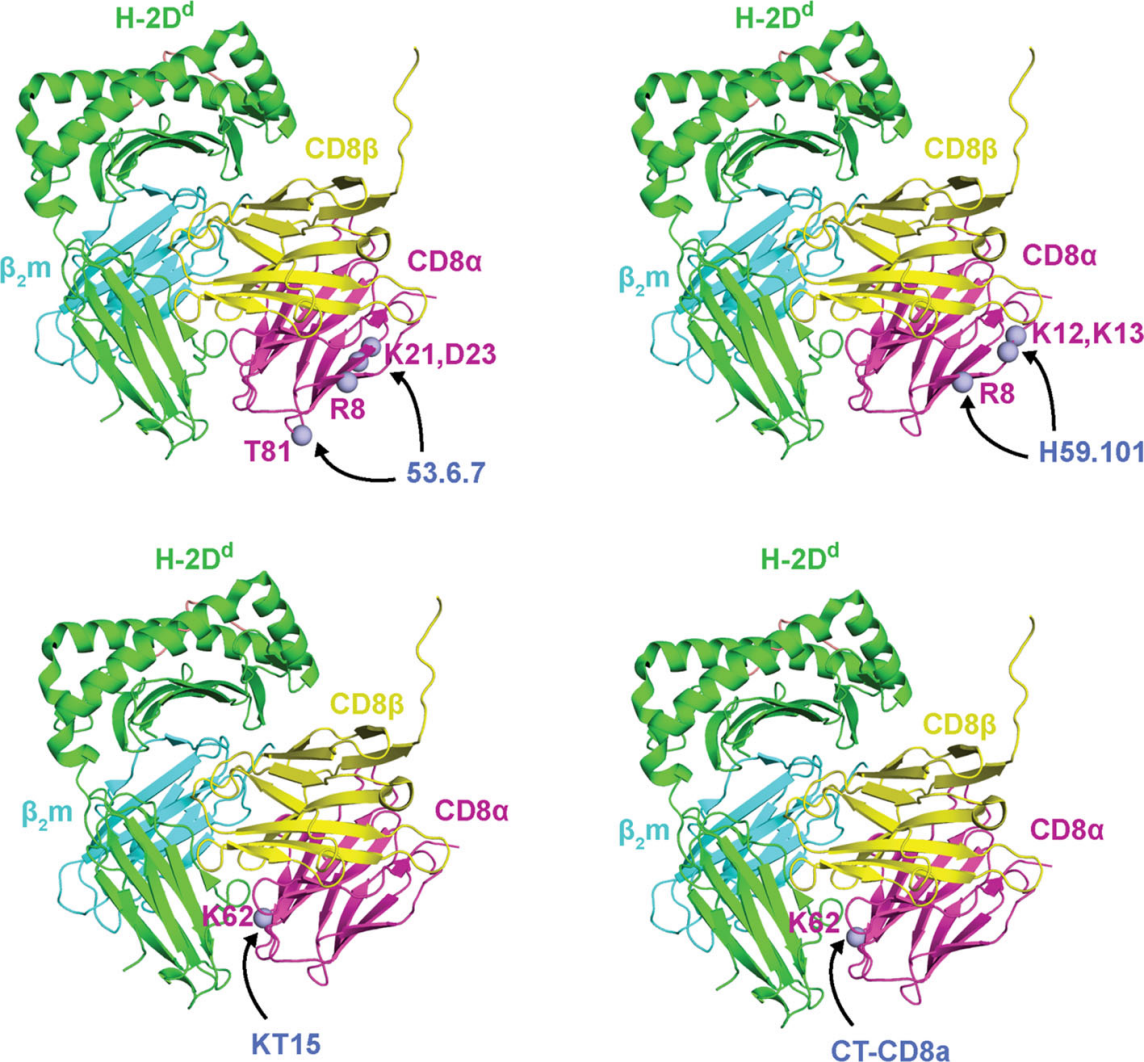
Site-directed mutagenesis identified CD8α epitopes recognized by anti-CD8 mAbs. CDα mutations that influence mAB binding are shown as light blue spheres. From top left to bottom right: mouse CDα mutations at residues R8, K21, D23, and T81 (ectodomain numbering) reduce binding to 53.6.7 antibody, mouse CDα mutations at residues R8, K12, and K13 (ectodomain numbering) reduce binding to H59.101 antibody, and mouse CDα mutations at residues K62 (ectodomain numbering) reduce binding to KT15 and CT-CD8a antibodies. As a reference, mutations are plotted onto CDα from mouse CD8αβ/H-2D^d^ (PDB ID 3DMM). MHC-I heavy chains are colored green, β2m light chains are colored cyan, peptide antigens are colored salmon, CD8α chains are colored magenta, CD8β chains are colored yellow, and the mABs names are colored blue. Data were derived from Devine et al. (52).

Several anti-CD8 mABs block CD8^+^ T cell activation through epitopes targeted on CD8α (*i.e.*, SK1, DK25, YTS 105.18, H59.101, OX-8, CT-CD8a, KT15, YTS 169.4) or CD8β (*i.e.*, YTS 156.7, 53.5.8, 2ST8.5H7) (10, 51, 173, 179) (**Table 2**). Some mABs block CD8^+^ T cell activation and maturation by mechanisms independent of the MHC-I/CD8 interaction. Interestingly, mAB binding with CD8 does not always abrogate CD8^+^ T cell signaling. For example, anti-CD8α mABs (53.6.7, OKT8) and anti-CD8β mAb (KT112, CT-CD8b) enhance CD8^+^ T cell signaling (10, 50, 52, 180) (**Table 2**). As for YTS 105.18 and YTS 156.7, most blocking anti-CD8 mABs appear to work by one of two mechanisms: *i)* sterically hindering CD8/MHC-I interactions, or *ii)* altering the topology of the immunological synapse relative to the T cell or antigen presenting cell membranes (10, 51, 173, 179). While structures of many of these CD8/mAB complexes are lacking, the general location of the epitopes recognized by some of these mABs has been mapped by site-directed mutagenesis to the CD8 ectodomain (52). Other mABs, such as the anti-CD8α MRC OX-8 antibody, have shown an ability to recognize the CD8 hinge region to carry out their function (54). Binding of blocking mABs KT15 and CT-CD8a was decreased by mutating CD8α residue K52 located on the CDR2 loop at the interface with the MHC-I, suggesting they sterically hinder CD8αβ/MHC-I interactions (**Figure 8**). Binding of blocking mAB H59.101 to CD8α was decreased by mutating CD8α residues R8, K12, and K13 located on the A strand, suggesting it does not sterically hinder CD8αβ/MHC-I interactions (**Figure 8**).

Less is known about the mechanisms driving anti-CD8 mAB enhancement of CD8^+^ T cell responses. However, they seem to enhance CD8αβ/MHC-I or CD8αα/MHC-I binding, through either stabilizing CD8/MHC-I complexes or influence signal transduction from the ectodomain to the cytoplasmic tail. For example, the inhibition of Lck reduces the enhancing effect of 53.6.7 antibody, suggesting that it functions by modulating signal transduction of CD8αβ ectodomain to the cytoplasmic tail (13, 52). The binding of enhancing mAB 53.6.7 to CD8α was decreased by mutating CD8α residues R8, K21, D23, and T81 located on the A, B, and D strands (52) (**Figure 8**). Another CD8^+^ T cell enhancing antibody, the anti-CD8α mAB OKT8, can induce cytokine release from several CD8^+^ T cells in the absence of specific MHC-I/TCR engagement, possibly through rearrangements in the immunological synapse topology (50). No structural information is available for the CD8αβ/OKT8 complex. Future studies should elucidate the atomic structure of CD8αβ and CD8αα with the monoclonal antibodies to provide mechanistic insights towards their application in immunomodulatory therapeutics.

## 5 Discussion

Several future directions are needed to move the utility of CD8 for immunomodulation into new horizons. First, relative to CD8αβ, the exact biological functions of CD8αα, and potentially CD8ββ, remain to be fully understood, especially in the context of non-conventional immune cell subsets. For example, in what cases does CD8 function as a co-stimulatory receptor versus a co-repressor? How do the differences in the structure of CD8α and CD8β regulate their function? Second, structural insights into important features of CD8 remain to be determined (*i.e*, hinge domain, transmembrane domain, cytoplasmic tails). Third, the binding affinities and structures of CD8αα and CD8αβ with a wider range of classical MHC-I alleles across different species, and non-classical MHC-I alleles (*i.e.*, CD1, T22, M10.5, MILL, HFE, FcRn), is required (181). This new structural and biophysical information will inform the potential for immunomodulation of for the broader class of MHC-like molecules by anti-CD8 mABs. Fourth, further details concerning the features of CD8 hinge and transmembrane domains that contribute to efficacy of CAR T cell signaling should be examined. Fifth, many anti-CD8 antibodies still require biophysical and structural characterization with CD8αα or CD8αβ to determine their binding epitopes and mechanisms of action (**Tables 1**, **2**). Finally, it is also likely that computational antibody design can be applied for the rational design of stable, tunable anti-CD8 mABs that target desired functional sites of CD8α or CD8β (182, 183) (**Figure 1D**). In all of the above cases, care must be taken since mABs can exhibit off-target effects or immunotoxicity, resulting in mAbs-induced adverse effects related to immunosuppression and hypersensitivity (184). Ultimately, we expect that in the coming years the CD8 co-receptor will emerge as one of the prime targets for immunomodulation.
